# Biomechanical considerations on a CT-based treatment-oriented classification in radius fractures

**DOI:** 10.1007/s00402-020-03405-7

**Published:** 2020-03-19

**Authors:** W. Hintringer, R. Rosenauer, Ch. Pezzei, S. Quadlbauer, J. Jurkowitsch, T. Keuchel, T. Hausner, M. Leixnering, H. Krimmer

**Affiliations:** 1 PK Döbling, Heiligenstädter Strasse 55-63, 1190 Vienna, Austria; 2 AUVA Trauma Hospital Lorenz Böhler, European Hand Trauma Center, Donaueschingenstrasse 13, 1200 Vienna, Austria; 3 Ludwig Boltzmann Institute for Experimental and Clinical Traumatology, AUVA Research Center, Donaueschingenstrasse 13, 1200 Vienna, Austria; 4 Austrian Cluster for Tissue Regeneration, 1200 Vienna, Austria; 5 Department for Orthopedic Surgery and Traumatology, Paracelsus Medical University, Strubergasse 21, 5020 Salzburg, Austria; 6 Hand Center Ravensburg, Elisabethenstraße 19, 88212 Ravensburg, Germany

**Keywords:** Distal radius fracture, Treatment-oriented classification, Key fragment, Implant selection, Biomechanics of the wrist

## Abstract

A wide range of different classifications exist for distal radius fractures (DRF). Most of them are based on plane X-rays and do not give us any information on how to treat these fractures. A biomechanical understanding of the mechanical forces underlying each fracture type is important to treat each injury specifically and ensure the optimal choice for stabilization. The main cause of DRFs are forces acting on the carpus and the radius as well as the position of the wrist in relation to the radius. Reconstructing the mechanism of the injury gives insight into which structures are involved, such as ruptured ligaments, bone fragments as well as the dislocated osteoligamentous units. This article attempts to define certain key fragments, which seem crucial to reduce and stabilize each type of DRF. Once the definition is established, an ideal implant can be selected to sufficiently maintain reduction of these key fragments. Additionally, the perfect approach is selected. By applying the following principles, the surgeon may be assisted in choosing the ideal form of treatment approach and implant selection.

## Introduction

The treatment options for DRFs have vastly improved over the years. Beginning with conservative treatment including closed reduction and plaster casts [1–8], K-wires were the first invasive method of stabilization. They were partly used in combination with external fixation. However, secondary dislocation still occurred [9–14], that necessitated correction and salvage procedures [15–27].

Later stabilization methods progressed from non-angular stable to angular stable plates, primarily using mono- then polyaxially angular stable screws. The first models to be introduced had one single row of distal screws, but were soon replaced by double row plates. Today, companies offer a wide range of specifically designed plates and screws to provide ideal stabilization for each fracture type. Arthroscopically assisted techniques broadened the technique spectrum especially when reducing intraarticular fractures [28–35]. Selecting the optimal choice from the different options available becomes difficult, especially for young surgeons with minor experience. Therefore, an enhanced biomechanical understanding of the different fracture types should facilitate the right decision for treatment.

This paper aims to provide a treatment-oriented concept for stabilizing DRFs based on a state-of-the-art fracture classification.

## Classifications of distal radius fractures

In the past, classifications were mainly based on plain X-rays. CT scans, 3D reconstructions, and 3D printing are useful diagnostic tools to enhance our understanding of these fractures and improve treatment options. Analyzing CT scans provided new findings, especially in intraarticular fractures, which were included in modern classifications [36].

Pechlaner [37] presented basic principles of fracture localization and formation using a device that produced fractures in fresh frozen cadavers. He showed that even in case of a dorsal extended wrist, palmar dislocated fractures are possible depending on the point of impact. His classification also included the importance of ligament insertion points in dislocated fractures, based on acting forces.

Mandziak et al. [38] demonstrated the correlation between fracture lines and the insertion points of the ligaments on the palmar and dorsal aspect of the radius.

Bain et al. [39] showed, that in most two-part fractures, recurring fracture lines can be found depending on the ligament insertions. He introduced the term “osteoligamentous unit”.

Brink and Rikli [24] presented a simplified classification based on four pillars, each possessing specific biomechanical functions and a special bearing to the dislocation mechanism. The critical fragment, that causes the shift of the carpus in different directions, was called the “key fragment”.

The main aim of this paper is to combine and modify these classifications, with an improved understanding of the biomechanics of the “key fragments”. Thereby, an opportunity to establish a treatment concept, to stabilize critical fragments using different types of internal fixation, should be possible. This procedure involves analyzing the primary plain X-rays (to estimate the grade of dislocation), the CT scans (to precisely define the key fragments and fractures lines) and 3D reconstructions or 3D models (for better fracture understanding and the bonus for teaching purposes).

## Biomechanical principles

**Fig. 1:**
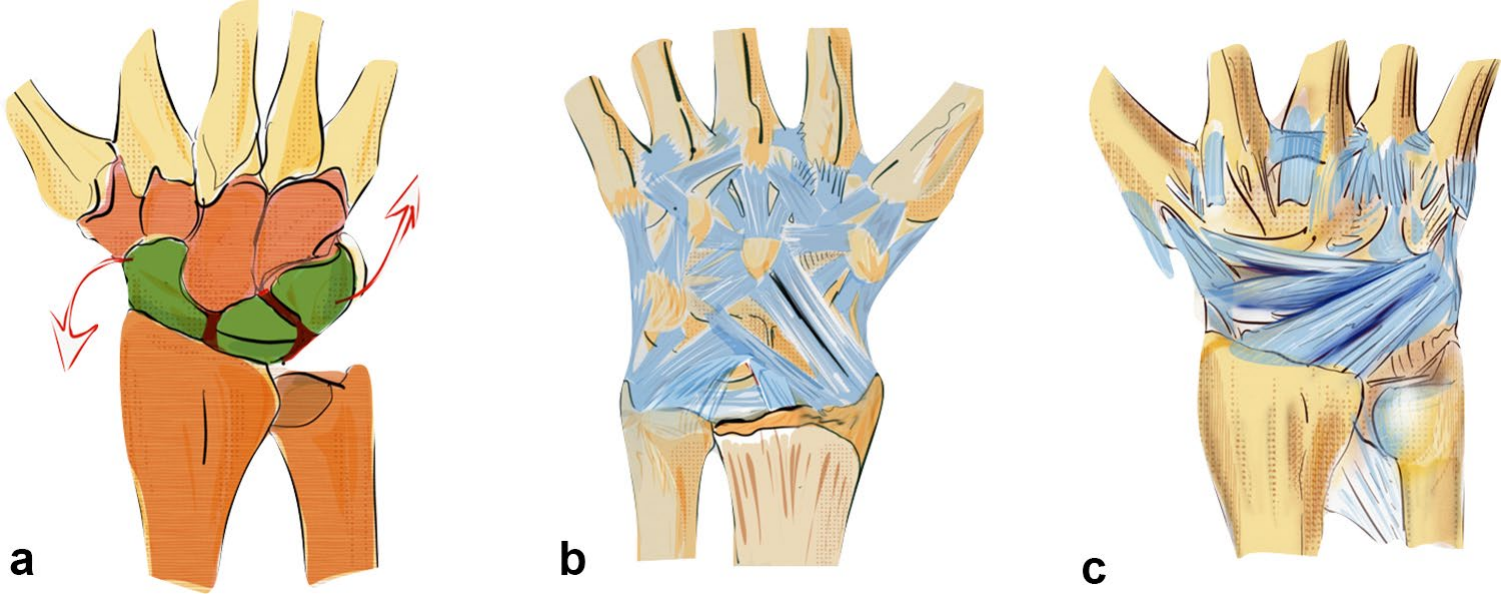
Prerequisite for normal working biomechanics is an intact bone stock. The first row acts as an intercalated segment between the two outer solid partners and is conjoined with short intrinsic ligaments (**a**). Palmar extrinsic ligaments: long extrinsic ligaments coordinate the movement between the carpal rows and hold the lunate in position in the center of the first row with a strong attachment (**b**). Dorsal extrinsic ligaments coordinate movement on the dorsal side and help to control the first carpal row (**c**)

The basic prerequisites for regular motion of the carpus are:

- Intact bone stock/radius and ulna.
- Intact intrinsic ligaments conjoin the proximal carpal row to a variable geometrical condyle versus the invariable proximal and distal partners.
- Intact extrinsic ligaments which coordinate the proximal row with radius and ulna against the distal carpal row, which acts as a monolith (see Fig. 1a) [40].

**Fig. 2:**
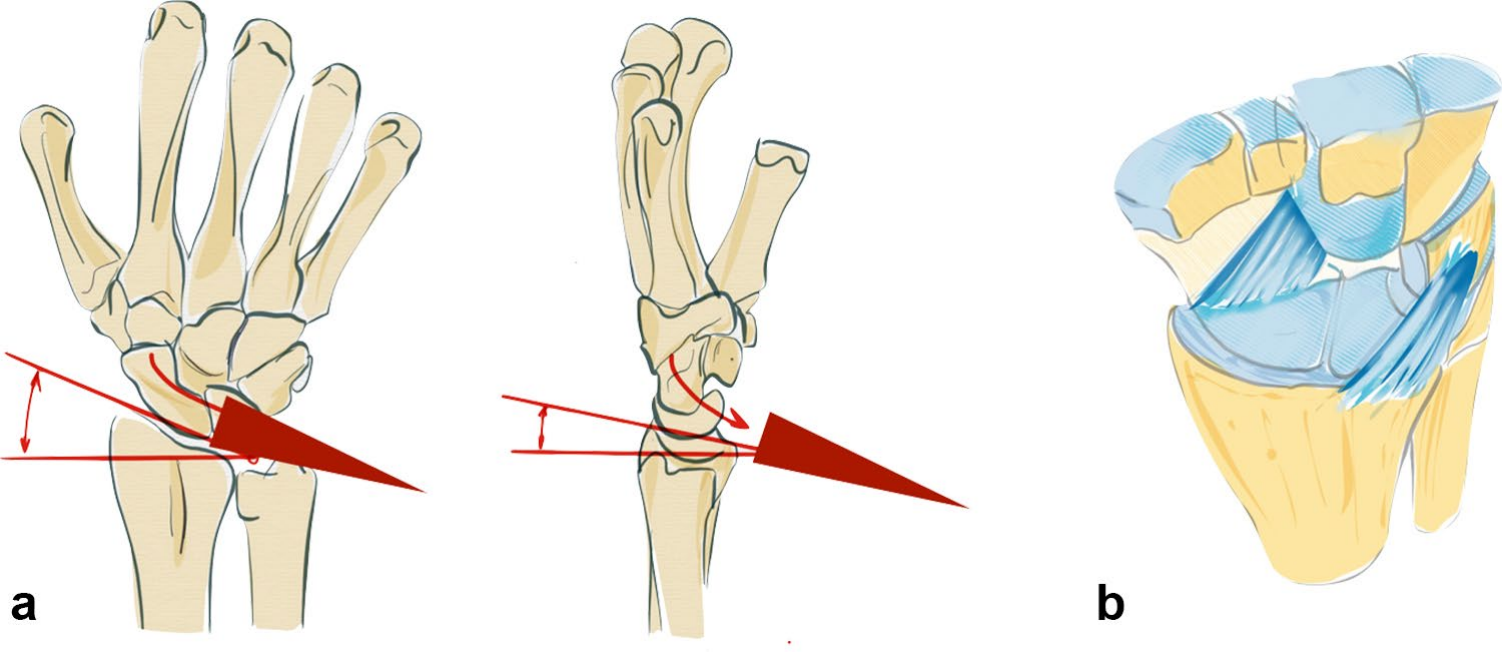
Tendency of the carpus to glide palmarly and ulnarly along the inclination of the radius to both sides (**a**) The palmar and dorsal extrinsic ligament form a sling around the carpus and hold it in position against dislocating forces (**b**)

The dorsal and palmar extrinsic ligaments compensate the tendency of the carpus to glide ulnarly and palmarly along the radial and palmar inclination (see Fig. 2a).

The so-called dorsal “v-ligaments” are on the dorsal aspect of the wrist (see Fig. 1c), the two proximal and distal “v-ligaments” are situated on the palmar aspect of the wrist and keep the carpus in position (see Fig. 1b, c).

**Fig. 3:**
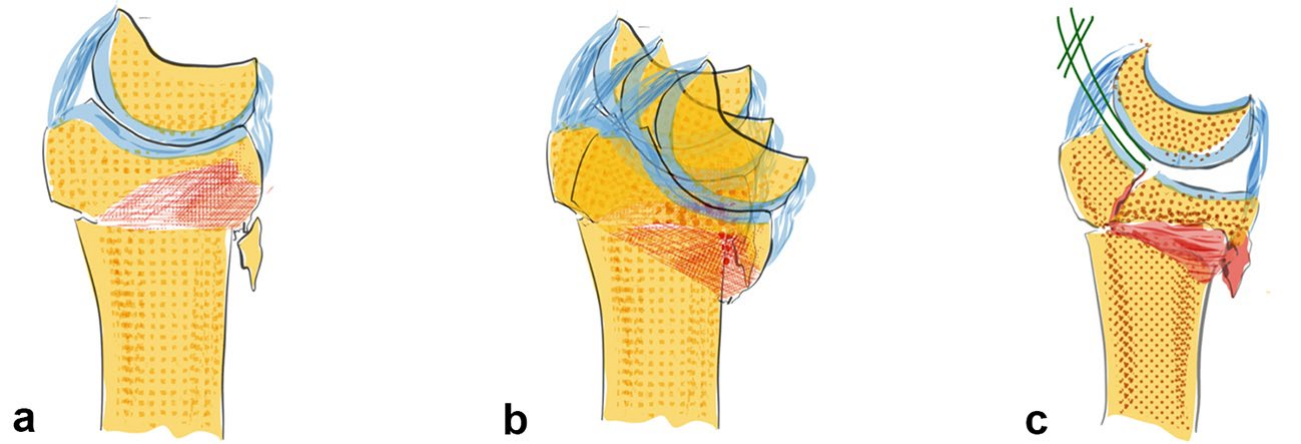
The greater the dorsal extending force, the greater the dorsal comminution zone (red area, **a**). The palmar ligaments act like a tension band, which additionally leads to a fracture of the palmar cortical bone (**b**). Increasing forces lead to intraarticular fractures and dorsal, radial or palmar key fragments (**c**)

Both the dorsal and palmar ligaments form a sling around the carpus which provides resistance against the acting forces (see Fig. 2b). The rather strong palmar ligaments support the proximal row like a belly tie and act against forces to the dorsal side like a tension band [41]. In case of trauma to the dorsally extended wrist, transmission forces act on the palmar ligaments. This either leads to a rupture of the palmar ligaments or if they remain intact a compression fracture on the dorsal aspect of the radius or also on the palmar side (see Fig. 3a–c) [42].

**Fig. 4:**
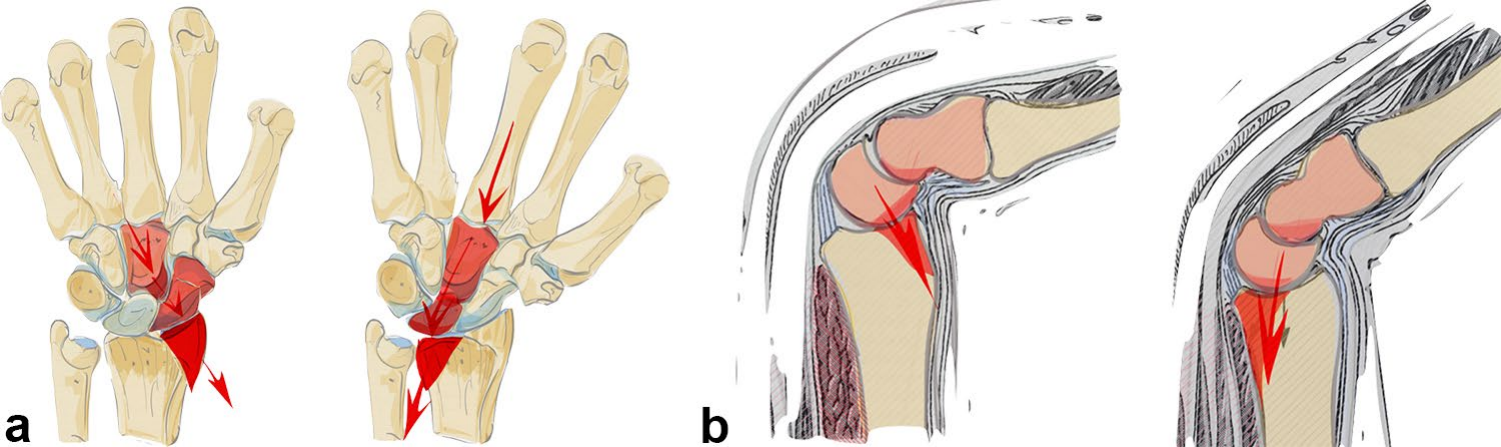
Depending on acting forces radial sided or ulnar sided fractures occur. In the first image, the acting force is transmitted via the capitate, scaphoid and finally the radial styloid, leading to a radial sided fracture. The second image demonstrates a transmission of the acting force through the capitate, lunate and sigmoid notch, leading to an ulnar sided fracture (**a**) A dorsal extended wrist does not necessarily lead to a dorsally dislocated fracture. Depending on the direction of the acting force, dorsal or palmar fractures may occur (**b**)

Depending on the direction of acting forces, radial sided or ulnar sided fractures can occur (see Fig. 4a). The direction of the force in relation to the position of the wrist on impact determines the fracture site, either a dorsal or palmar fracture (see Fig. 4b).

**Fig. 5:**
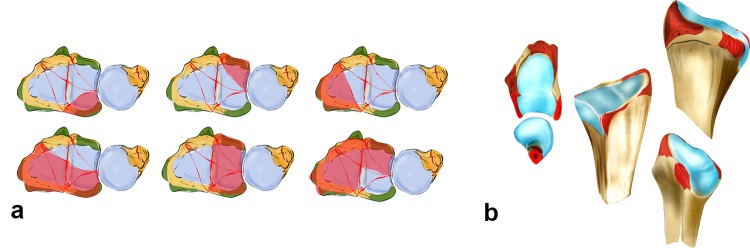
In partial intra-articular fractures, six different patterns can be observed. At least one corner remains intact and in continuity with the shaft (**a**). The origins of the extrinsic ligaments are shown, which seem to reinforce the bone (**b**)

The basic factors that cause DRF include acting forces, the position of the wrist and the resistance of the ligaments. Specific fracture types arise from the interaction between these parameters (see Fig. 5a). The question arises whether the fracture lines show a distinct or randomized pattern. Fracture lines seem to occur between the insertions of the extrinsic ligaments (see Fig. 5b). These ligaments appear to reinforce the bone at their origins. Fracture patterns in two-part fractures generally occur in the area between the ligamentous zones. Intraarticular fractures show six different fracture patterns. At least, one corner remains intact with the shaft (see Fig. 5a). From a biomechanical point of view, these bone–ligament fragments form a unit and tend to dislocate in different directions depending on their ligamentous attachment sites.

## Key type fractures

### Radial key fragment

On the dorsal side the radiotriquetral ligament and from palmar aspect the radiolunate and radiocapitate ligament form a sling around the carpus that reinforces the styloid against acting forces [43].

**Fig. 6:**
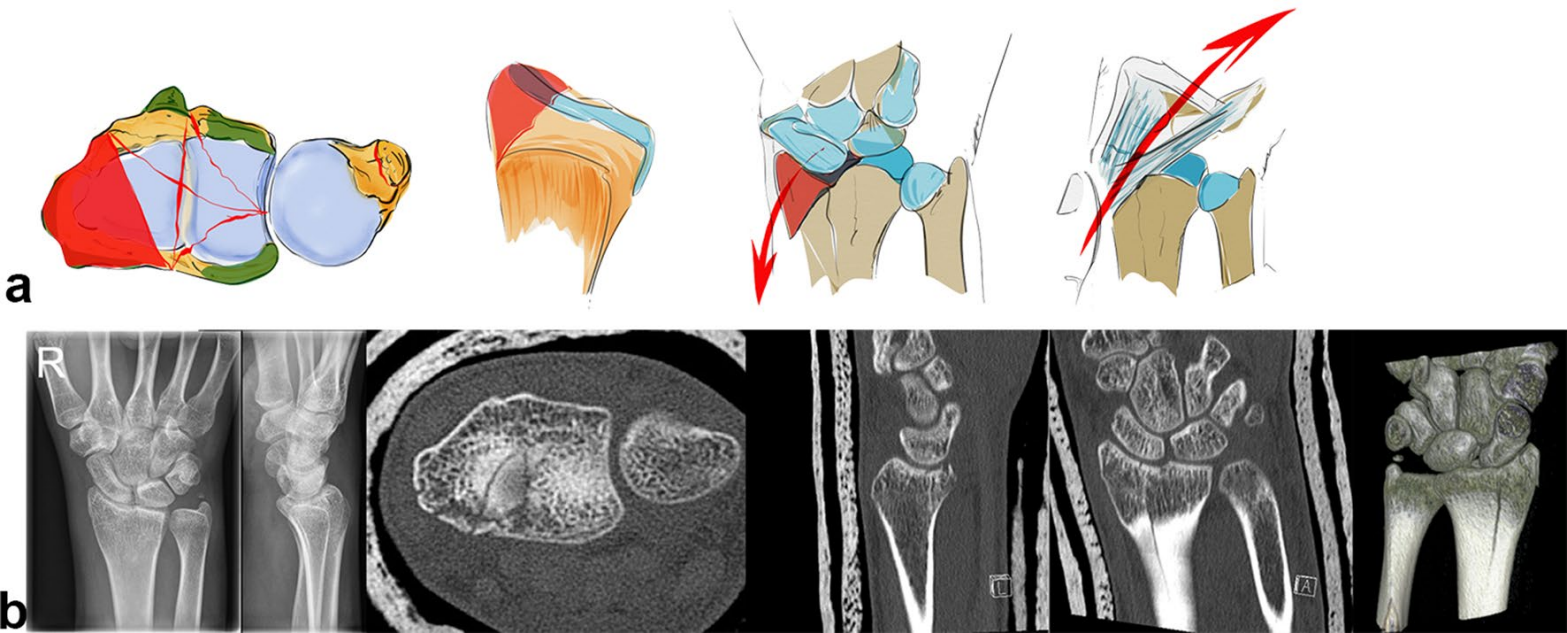
A schematic illustration showing a radial key type fragment with dislocation of the osteoligamentous unit to the radial side (**a**). Example of a radial key type fragment. The plain X-rays show the relatively low grade of dislocation. However, the CT scan delineates the long fracture line into the radius shaft (**b**)

A radial acting force is directed along the capitate, scaphoid and lunate onto the styloid process, which fractures along the insertion of the ligaments and dislocates in a radial palmar or dorsal direction (see Fig. 6a). The styloid process and the lunate form a unit linked by their ligaments. In case of trauma, the capitate protrudes between the lunate and the scaphoid, leading to a rupture of the scapholunate ligament. The carpus tends to subluxate radially (see Fig. 6b), due to the ligamentous attachments. Reduction can be achieved by ligamentotaxis.

### Palmar key fragment

**Fig. 7:**
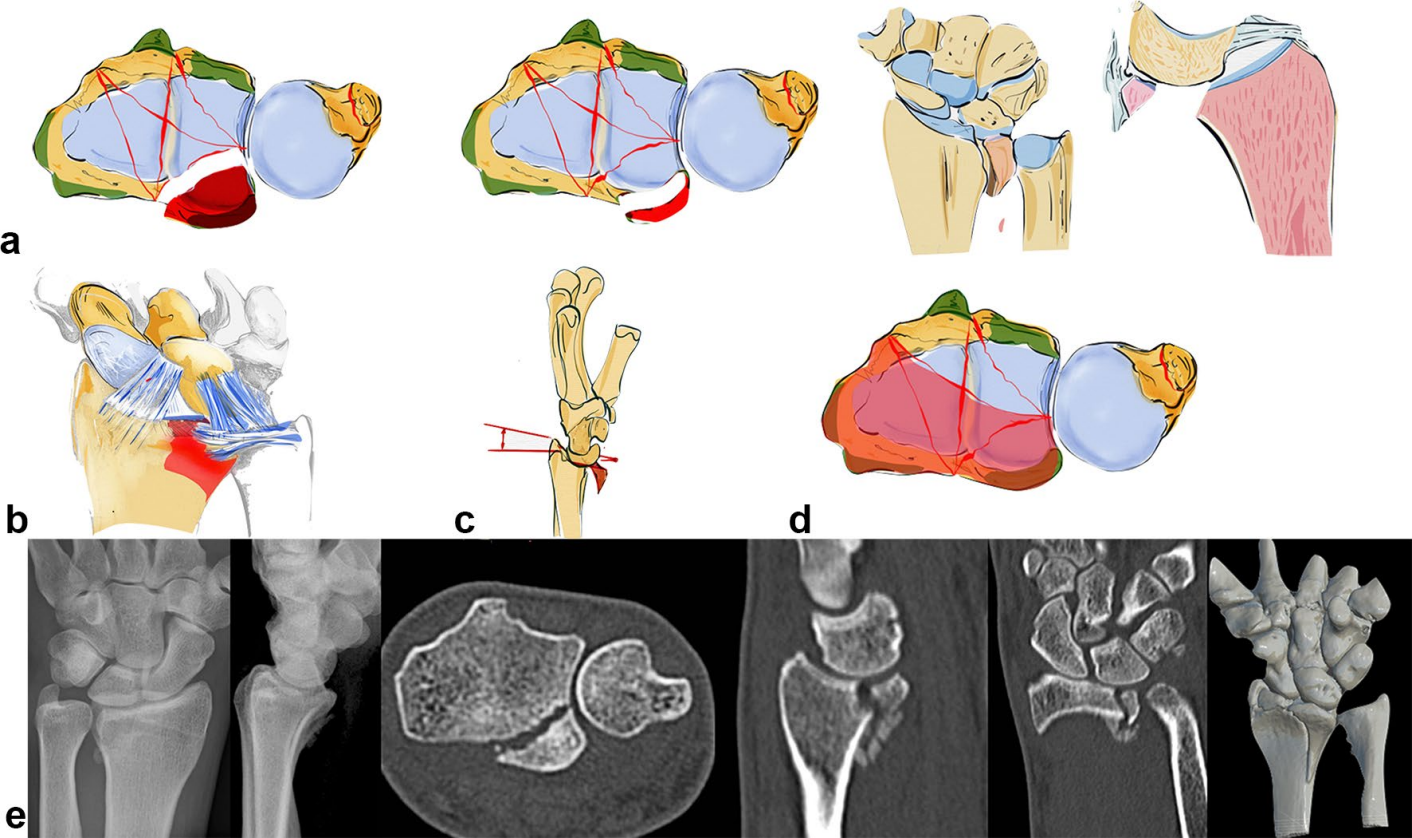
Palmar key type: the palmar ulnar osteoligamentous unit can either be a large or smaller rim fragment. Dislocation can occur in a palmar direction with the dorsal ligaments remaining intact (**a**). Palmar ulnar fragment: origin of important radioulnar and ulnocarpal ligaments (**b**). The osteoligamentous unit dislocates in a palmar direction (**c**). In extreme cases, a complete ulnar to radial palmar fragment is possible (**d**). Small ulnar palmar fragments can be easily overlooked on plain X-rays, axial CT scans however show this fragment best (**e**)

A palmar acting force leads to a fracture of the palmar cortical bone (see Fig. 7e) with depression of the palmar aspect of the radius or a palmarly dislocated fracture with a smaller or larger fragment (see Fig. 7a–c). This highly depends on the position of the dorsally extended wrist. An isolated fracture of an ulnar rim fragment is possible or alternatively the palmar fracture extends from the ulnar to the radial side (see Fig. 7d) [37].

Due to the fact that the palmar v-ligaments insert into these palmar fragments and support the proximal row like a belly tie, the whole carpus tends to dislocate in a palmar direction in case of a fracture of this osteoligamentous unit. The palmar ulnar fragment is the origin of the ulnocarpal and the palmar radioulnar ligaments which are the main stabilizers of the distal radioulnar and the ulnocarpal joint (see Fig. 7b). If these ligaments are impaired, the radiocarpal and radioulnar joint is destabilized.

Furthermore, palmar fractures with smaller fragments the so-called rim fragments are often overlooked and tend to show a higher grade of instability [44]. Beyond these bony injuries, accessory ligamentous lesions are possible.

### Dorsal key fragment

**Fig. 8:**
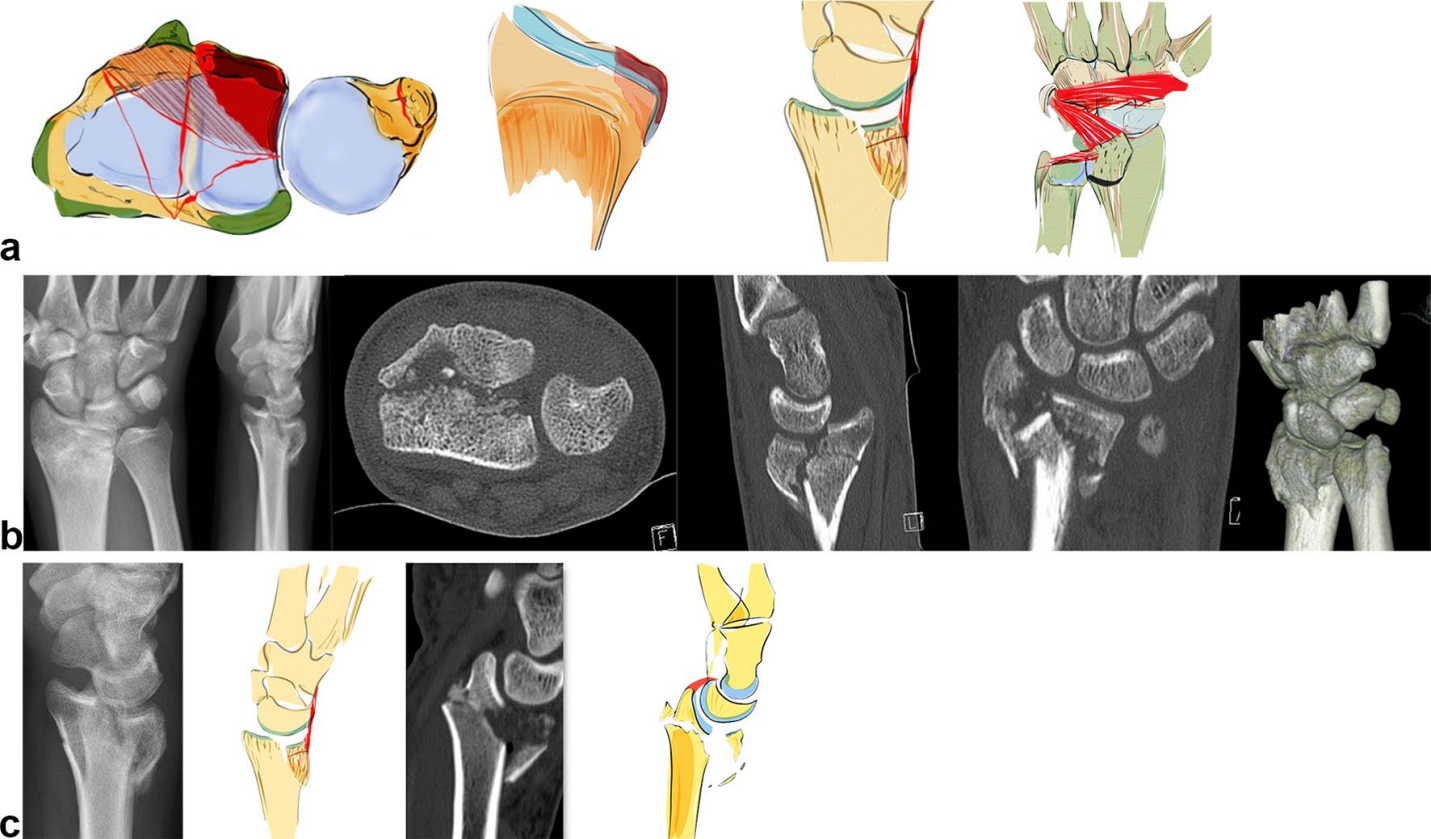
Dorsal key fragment: carpus dislocates with the key fragment to the dorsal side with dorsal extrinsic insertions. The palmar ligaments are ruptured (**a**). The plain X-rays show the grade of dislocation in a dorsal direction. On the CT scans, however, an additional depression of the articular surface, a step-off of the sigmoid notch and the dorsal key fragment is visible (**b**). A dorsal fragment does not necessarily have to be a key fragment. If the palmar ligaments are ruptured and the carpus and the dorsal fragment dislocate as a unit, the dorsal fragment has to be fixed first, as seen on the left two images. If, despite dorsal dislocation, the volar fragment is still attached to the palmar ligamentous apparatus (parallel articular lines between lunate and palmar articular surface), this palmar key fragment, comprising the osteoligamentous unit, requires special attention and has to be fixed first as seen on the right two images (**c**)

Acting forces exerted in a dorsal direction not only cause radius fractures on its dorsal aspect but also tend to dislocate the carpus in a dorsal direction (see Fig. 8b). These fractures occur either ulnarly or along the entire dorsal surface of the joint (see Fig. 8a). The radiotriquetral ligament and the dorsal edge fragment form an osteoligamentous unit and dislocate together to the dorsal side. Partial dislocation is possible when only fragments on the ulnar aspect of the dorsal aspect of the radius occur. Due to the displacement of the carpus towards the dorsal side rupture of the palmar ligaments with or without bony avulsion injuries is fairly common.

The carpus can also dislocate in dorsal direction without a key fragment. This fracture type still has a ligamentous connection to the palmar lip of the radius even when the carpus is dislocated in a dorsal direction. A parallel dislocation of the lunate and palmar fragment can be observed in the CT scan. This is an indication of the preserved osteoligamentous unit. The key principle is to stabilize this palmar osteoligamentous unit to ensure adequate reduction and stabilization of this fracture type (see Fig. 8c).

### Central key fragment

**Fig. 9:**
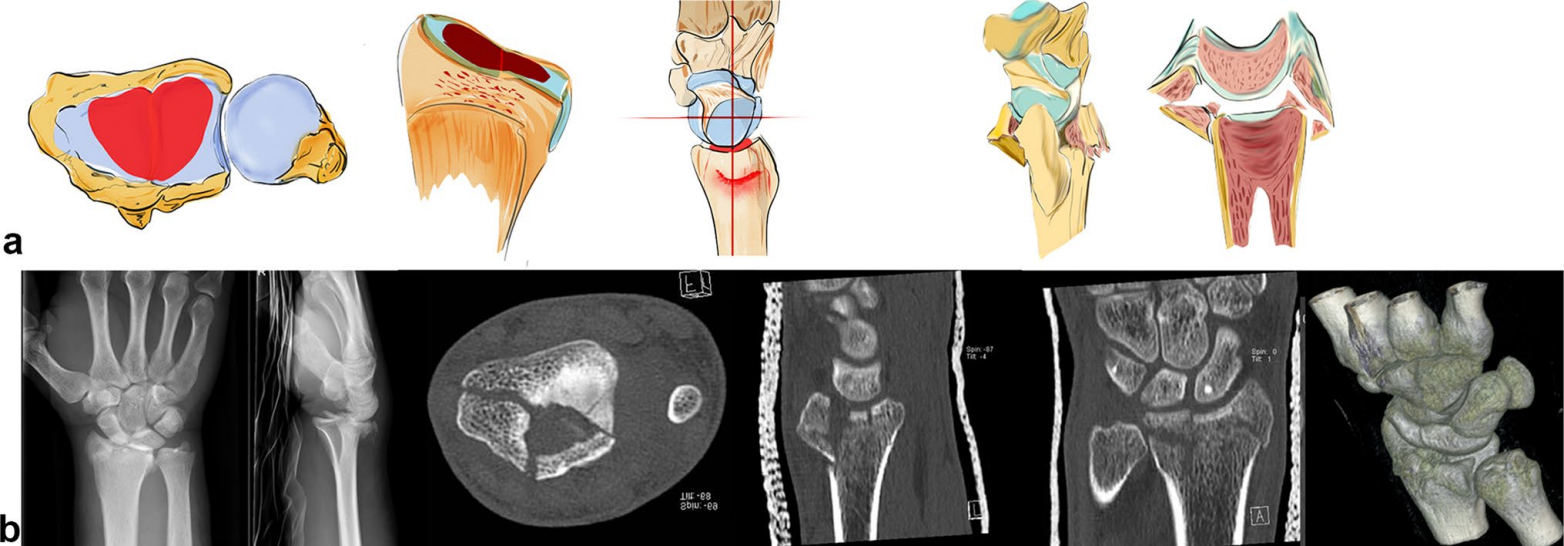
A central impression can occur with or without a dorsal or palmar fracture of the cortical bone. In these cases, the advantages of the CT scan are evident (**a** + **b**)

Axial acting forces can cause an isolated central depression of the articular surface or bursting fractures comprising both dorsal and palmar fragments (see Fig. 9b). This central key fragment has no ligamentous connection to the shaft or the carpus. Sometimes, it is only slightly depressed under the articular surface and easily overlooked, especially in plain X-rays. If this central fragment is impacted deeper into the radius shaft, both the dorsal and palmar cortical bone, on which the stabilizing ligaments are attached, open up like a tulip (see Fig. 9a).

## Non-key type fractures

### Distal shear fracture

**Fig. 10:**
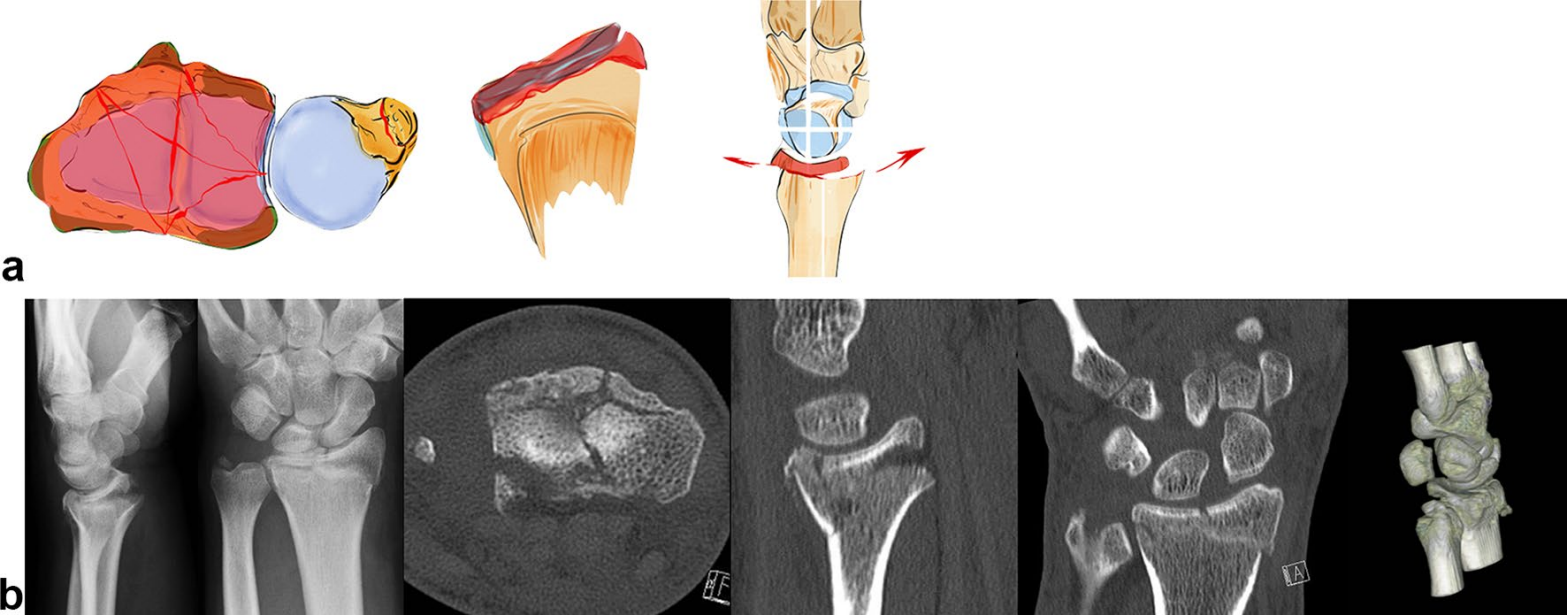
As can be seen on the CT scans, these distal shear fractures cause the fragments to float on the shaft of the radius like ice splinters. These are bony avulsions of all ligamentous insertions dorsal and palmar. Central shear fragments are without any ligamentous insertions. Since the fragments are extremely thin, it is difficult to grasp them and stabilize them sufficiently (**a** + **b**)

Tangentially exerted forces cause the carpus to dislocate dorsally or palmarly in the radiocarpal joint. The joint surface is sheared off in small fragments (see Fig. 10b). Depending on the direction of dislocation, the ligaments are attached dorsally or palmarly onto these tiny little fragments (see Fig. 10a).

Central parts can also be sheared off additionally with no contact to the radius shaft. These fractures are particularly unstable in all directions due to the complete detachment of their ligamentous insertions. Because these fragments are difficult to grasp, these fractures have a higher degree of instability.

### Three-part fractures

**Fig. 11:**
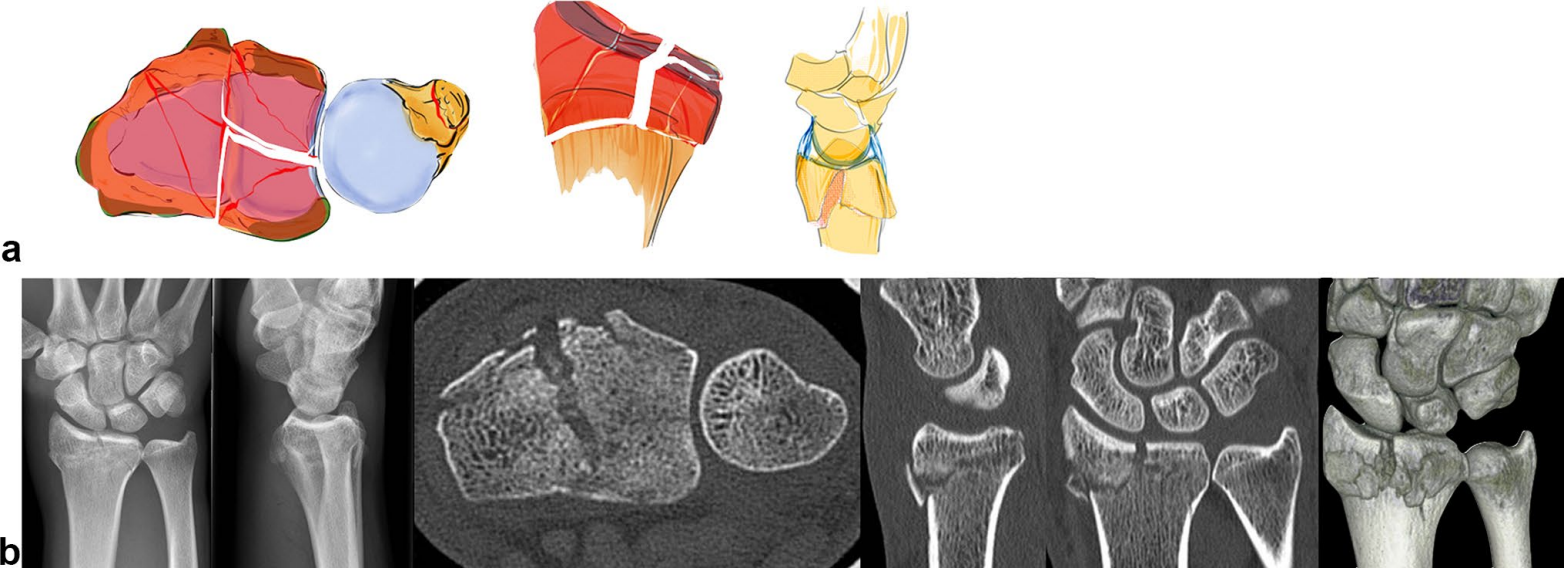
In these three-part fractures, depending on the insertion of the important extrinsic and intrinsic ligaments, these three key fragments must be reduced to restore stability. These are a combination of a radial, ulnar and dorso-ulnar key type fractures (**a** + **b**)

This fracture is a combination of a radial, a palmar ulnar and dorsal ulnar fragment (see Fig. 11a, b). Each fragment is the insertion point of important extrinsic ligaments that hold the carpus in position. These three osteoligamentous units are to be seen as equally important key fragments. Even though they have no connection to the shaft, repair is essential to maintain stability of the first carpal row against radius and ulna.

### Comminution fractures

**Fig. 12:**
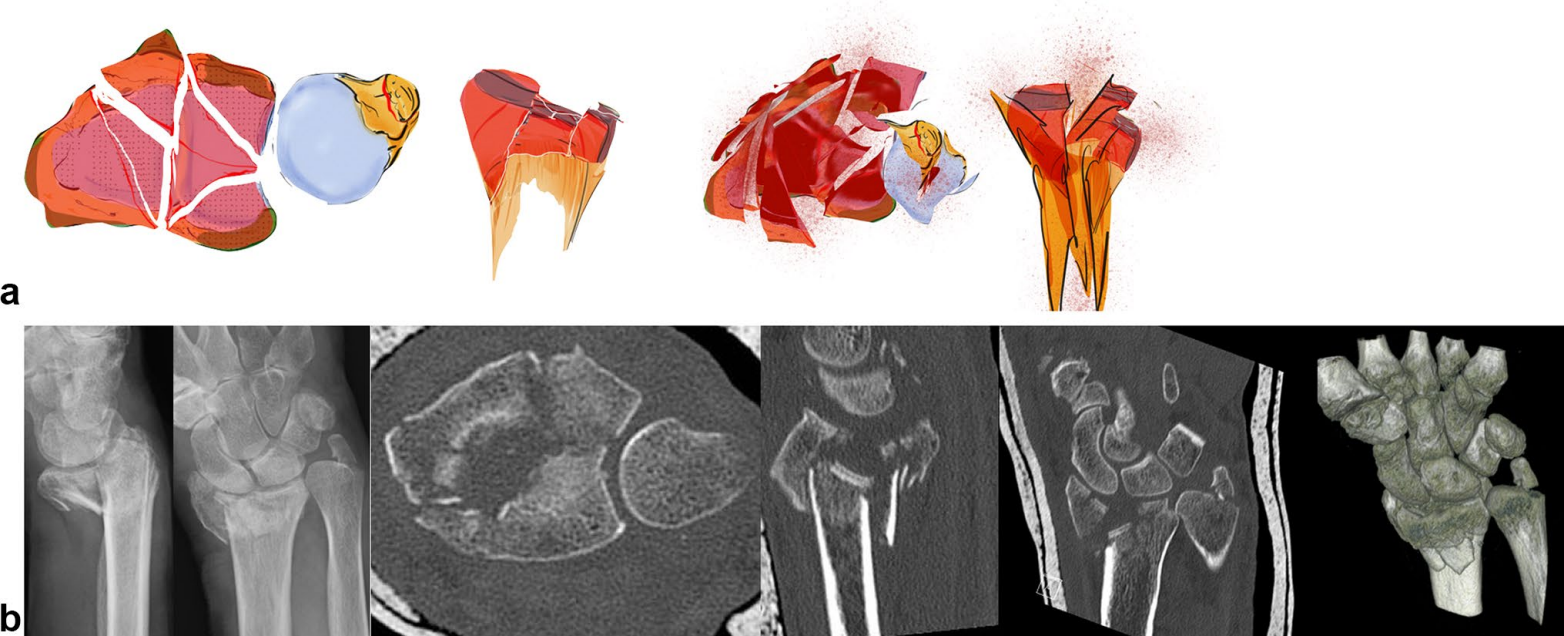
A comminution fracture consists of many small fracture elements which can neither be grasped or stabilized individually. In these cases, there is no addressable osteoligamentous unit (**a** + **b**)

In addition to these key type fractures, random fracture types with complete destruction of the radius joint surface occur (see Fig. 12a). The articular fragments float freely like ice floes over a metaphyseal comminution zone (see Fig. 12b). The ligamentous connection to these fragments cannot be addressed. These fractures have a high degree of instability and they tend to dislocate in any direction and impact in an axial direction.

## How to classify a fracture

To correctly classify fractures, X-rays in two planes and CT images are necessary. For difficult intraarticular fractures, 3D reconstructions are useful. 3D printing of fractures seems to be a valuable teaching tool and also assists in plate fitting. However, for reconstructive osteotomies, 3D images are imperative.

- Plain X-rays present an overall picture of the fracture including the main axes of dislocation.
- CT scans show the extent of the articular fracture in particular. First, the axial image should be seen and examined, because the position of the fragments in the sigmoid notch can be assessed. Together with the other two planes, a complete picture of the three-dimensional extent of the fracture is attained.
- Our three-dimensional imagery can be augmented by a 3D reconstruction.

## Reflections to find the ideal approach and type of implant

From the large number of implants available on the market, it seems crucial to consider which plate type would be most suitable to stabilize a specific fracture type, with regard to economic considerations—not every fracture type necessarily requires the most expensive treatment [45].

The first step is to determine the correct approach and to assess subsequent measures necessary, to prevent secondary dislocation of the carpus. This seems to be more important than a perfect reduction [46]. Most modern plates are polyaxially angular stable and can stabilize the distal fracture fragments with two rows of screws. Nevertheless, there are important aspects in the differing shapes of the plates that are generally unknown. The radially longer and more distally reaching plates, which have the advantage of grasping very distal fragments radially, do not consider the Watershade concept. The so-called Watershade plates are ulnarly longer and have to be positioned proximal to the Watershade line. They do not compromise the flexor tendons but offer only limited possibilities to grasp and stabilize the very distal fracture elements [47]. For palmar ulnar fragments, there are special plates from different manufacturers, specially designed to grasp very far distally placed ulnar fragments [48]. For the treatment of single fragments, cannulated self-tapping screws are becoming increasingly popular, especially in minimal invasive arthroscopically assisted methods.

The “single use sets” concept keeps the implant stock to a minimum, therefore preoperative planning of the procedure by the surgeon is essential to ensure that the specific implants for the osteosynthesis are in fact available for a special fracture type.

## Treatment options for different fracture types

Once the classification has been established, recognition of the key type will facilitate the ideal treatment options (see Table 1).

**Table 1:** Overview of the treatment, implant selection and approach to distal radius fractures depending on the fracture type and degree of the dislocation

Fracture type	Type of implant	Recommended approach
Metaphyseal fracture, dislocation less than 15° dorsal and 10° radial	Closed reduction, forearm cast for 4 weeks	
Metaphyseal fractures, dorsal dislocation more than 15° dorsal and 10° radial	Simple fracture plates Watershade plates Minimally invasive plates	Palmar approach Minimal invasive if possible
Metaphyseal fractures even with only slight palmar dislocation. Tendency to palmar dislocation	Simple fracture plates Watershade plates Minimally invasive plates	Palmar approach Minimal invasive if possible
Radial key fragment	Screws Radially oriented plates Double-headed screws	Radial or palmar approach, arthroscopically assisted
Dorsal key fragment	Dorsal buttress plates possibly in addition to palmar plates Dorsal plates Transfixation of the carpus if necessary	Dorsal or palmar approach, arthroscopically assisted
Palmar key fragment	Ulnar oriented plates Watershade plates Ulnar special plates Hook plates and screws Transfixation of the carpus if necessary	Palmar approach Arthroscopically assisted
Central key fragment	Fracture plates, Watershade plates, Cancellous/or artificial bone grafting in large defects	Arthroscopically assisted Palmar closed indirect reduction Dorsal open reduction
Articular comminuted fractures	Plates with maximum number of polyaxially angular stable screws with 2 distal rows Double plating if necessary	Palmar approach Additional dorsal approach in case of double plating, arthroscopically assisted if possible
Articular comminuted fractures extending far into the shaft No identifiable fragments to be stabilized	External fixator Spanning plate	Dorsal

### Radial key type

**Fig. 13:**
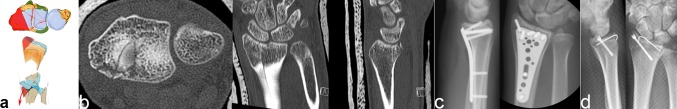
Schematic illustration of a radial key type fragment (**a**). The CT scan shows the long fracture line into the radial shaft (**b**). Palmar plating with implants which are radially longer and ulnarly shorter are suitable to stabilize such fractures (**c**), Cannulated screws are possible if the surgery is arthroscopically assisted (**d**)

Radial key type fractures are best treated with radial oriented plates (see Fig. 13a–c). These plates have a longer radial and shorter ulnar border and are the mirror image of the ulnar oriented watershed plates.

These radial plates can be mounted very far distally, thereby grasping fragments that cannot be reached by watershed plates. The main disadvantage of this type of plate is the potential damage to the flexor tendons, depending on the plate position. In addition, the plate needs to be removed after fracture healing. A good method for treating single styloid fractures without depression of the articular surface are K-wire guided, cannulated, double headed screws, especially if the surgery is done with arthroscopic assistance (see Fig. 13d).

*Access* Primarily from the palmar side. Radial mounted plates in the first extensor compartment are being replaced by polyaxially locking plates with two rows that can also grasp these fragments from the palmar side.

### Palmar key type fractures

**Fig. 14:**
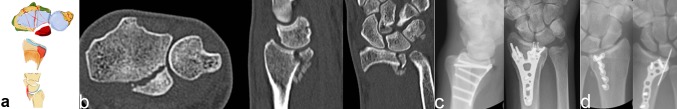
Schematic illustration of an ulnar key type fragment (**a**). The CT scan shows the palmar dislocation and the small size of the fragment (**b**). The so-called Watershade plates which can be mounted very far distally and ulnarly and special plates for the ulnar side have to be used to stabilize these fragments (**c** + **d**)

**Fig. 15:**
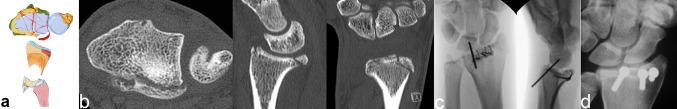
Schematic illustration of an ulnar palmar rim fragment (**a**). Only the CT scan visualizes these small fragments (**b**). Small hook plates (**c**), larger plates with attached hooks, or screws (**d**) can be used to fix these fragments

**Fig. 16:**
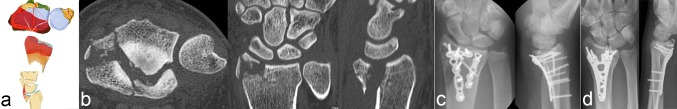
Schematic illustration of a palmar radioulnar key type fragment (**a**). The fragment is easily identified in the axial CT scan (**b**). Long distal frame plates, fracture-specific plates (**c**) or FPL plates (**d**) can be used in these cases

Similarly, palmar plates should be used to treat palmar key type fractures. In the case of a palmar key fragment, one must differentiate between the fragment sites, if it is only ulnarly (see Fig. 14), if there is a rim fragment (see Fig. 15) or if it also extends to the radial side (see Fig. 16).

*Access* palmar approach.

#### Palmar ulnar type

The so-called Watershade plates are optimal for ulnar sided palmar fragments as they can be mounted very far ulnarly as well as distally (see Fig. 14c). They therefore do not compromise the flexor tendons on the radial side. In addition, special plates for isolated stabilization of the lunate facet are available. These very narrow plates minimize contact to the flexor tendons but can only be used for limited indications (see Fig. 14d).

#### Palmar rim fragment

If the palmar fragments are too small to be adequately stabilized by a single plate, alternatives such as small hook plates (see Fig. 15c), screws (see Fig. 15d) and special plates with attached hooks can be used to grasp these rim fragments, thereby increasing stability and preventing palmar dislocation.

#### Palmar radio ulnar type

If the palmar fragment extends as far as the radial aspect, a wider distal plate has to be used to incorporate these fragments.

Special plates are available with two separated arms (see Fig. 16d). The space between the two arms is intended for the flexor pollicis longus tendon. Theoretically, the tendon runs in this space and pressure on the tendon is reduced to a minimum. The Soong concept can be neglected when using these implants. Alternatively, special frame plates, mounted far distally, can be used. However, an early plate removal has to be planned if they are placed distally to the Watershed line (see Fig. 16c).

Once the palmar fragments have been stabilized, an inspection for any remaining palmar instability must be performed as an accessory ligamentous lesion is likely. In this case, the carpus requires temporary transfixation to the radius in a neutral position with one or two K-wires to prevent secondary dislocation. These K-wires have to be removed after 6 weeks, when the cast is removed.

### Dorsal key type fractures

**Fig. 17:**
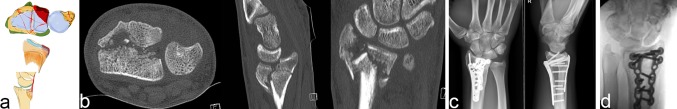
Schematic illustration of a dorsal key type fragment (**a**). The CT scan shows the dorsal dislocation of the dorsal key fragment together with the carpus as an osteoligamentous unit (**b**). If there is an additional palmar fracture, then double plating both palmar and dorsal is necessary. (**c**) Single dorsal fragments should be stabilized from dorsal aspect (**d**)

Dorsal key type fractures should be treated from the dorsal side especially if the dorsal fragment cannot be correctly reduced from the palmar side and are too small for fixation from the palmar side (see Fig. 17d). If the fracture also includes palmar fragments, then a combined palmar and dorsal approach is necessary (see Fig. 17c).

The dorsal approach can be done selectively over the dislocated dorsal key fragment, especially if the fragment is dorso-ulnar. In this case, small buttress plates are useful. The irritation to the extensor tendons is the main disadvantage in all dorsal stabilizations. However, the use of advanced low-profile plates is recommended, as they can significantly reduce this problem [49, 50] (see Fig. 17d).

A palmar plate may also be used if the isolated, large dorsal key fragment can be reduced indirectly and the palmar screws ensure secure fixation. These are mostly ulnar-dorsal sigmoid notch fragments and large enough to be grasped from the palmar side.

*Access* dorsal limited or dorsal wide exposure depending on the fracture type. In limited situations indirect reduction from palmar with palmar plate fixation.

**Fig. 18:**
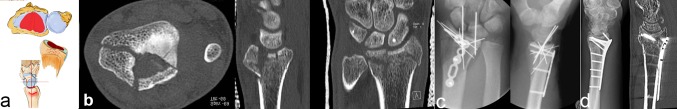
Schematic illustration of a central impaction (**a**). The type of fracture is easily identified in the axial CT scan (**b**). Small palmar plates with (**c**) or without (**d**) K-wires can be used to stabilize these fractures. Transfixation with K-wires is necessary if the carpus still tends to dislocate dorsally or palmarly after stabilizing the fragments

### Central key type fractures

Central depressions of the articular surface are sometimes difficult to detect. If the depression is centrally confined and the palmar and dorsal cortical bone remain intact, then CT scans are best to determine the extent of depression. Arthroscopically assisted procedures are the best choice for treating these fragments.

Occasionally indirect reduction under X-ray intensifier with palmar plating using polyaxially angle stable plates including two distal rows to support the articular surface is feasible. The depressed area is corrected by a hole drilled into the palmar cortical bone. If the cortical bones fracture in a tulip-like fashion under the central depression, then a dorsal approach generally offers the best access to the radiocarpal joint. In this case, dorsal plating is a good choice. K-wires are optional (see Fig. 18c, d).

*Access* dorsal limited or dorsal wide exposure depending on the fracture type. In selected situations indirect reduction from palmar and palmar plate fixation.

### Distal shear fractures

**Fig. 19:**
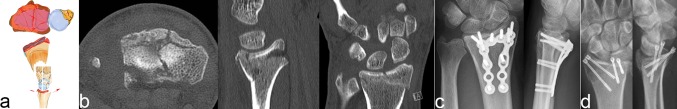
Schematic illustration of a distal shear fracture (**a**). CT scan shows the small fragments (**b**), which are difficult to grasp. Far distally placed frame plates (**c**) or screws (**d**) can be used in these cases

Distal shear fractures are comparable to a ligamentous radiocarpal dislocation. In this case, the shear fragments have no contact to the intact radius shaft. The articular surface fractures with small fragments occur very far distally and include the palmar and dorsal ligamentous insertions. These fragments are very difficult to stabilize, therefore plates which can be placed very far distally are necessary. Frame plates with a dorsal or palmar approach or single screws depending on the type of fracture can be used (see Fig. 19c, d). If sufficient stabilization cannot be achieved, then temporary fixation of the carpus is necessary. Sometimes, spanning plates are used.

*Access* Depending on the direction of dislocation, palmar or dorsal access is chosen.

### Three-part fractures

**Fig. 20:**
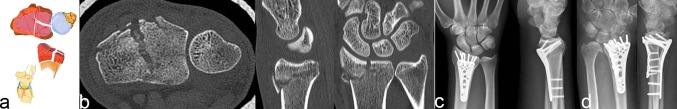
Schematic illustration of a three-part fracture (**a**). This type of fracture can be identiied best in the axial CT scan (**b**). Palmarly placed watershed plates (**c**) can be used in these cases, double-plating can be performed if necessary (**d**). (Figure 20d was taken from Quadlbauer et al. [51] and reproduced with permission from Springer)

Three-part fractures are usually accessed by a palmar approach. Particular attention must be paid to correctly reduce the sigmoid notch, as it is not visible in this approach. Occasionally, an additional dorsal approach is necessary. Watershade plates stabilize these fractures best and reduce the risk of tendon damage (Fig. 20).

*Access* palmar approach (limited dorsal approach).

### Comminuted fractures

The entire articular surface breaks into separate pieces and has no contact to the radius shaft. A comminution zone appears in the metaphyseal area. The articular surface looks like floating ice. As long as the fragments are identifiable, they can be treated individually and fracture specific. Polyaxially, angle stable plates including many holes with two rows are the best option. In the first row, the screws are positioned under the palmar part of the articular surface and in the second row under the dorsal part. Preferably the biggest fragments should be grasped by screws, but if that is not possible then the screws should be placed in a randomized grid-like fashion under the articular surface.

**Fig. 21:**
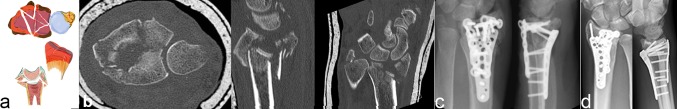
Schematic illustration of a comminution fracture (**a**). The CT scan visualizes the intraarticular step-off of several small fracture parts (**b**). Combined palmar and dorsal approach and double plating from palmar and dorsal is an option (**c** + **d**)

**Fig. 22:**
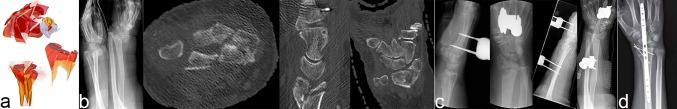
Schematic illustration of a severe comminuted fracture (**a**). The CT scan (after applying an external fixator) shows the complete destruction of the articular surface (**b**). In these high-energy traumas, DRF are temporarily stabilized with external fixator (**c**) or a spanning plate (**d**), as the patient’s condition needs to be the primary concern. The final/second treatment of DRF then takes place under ideal circumstances

A palmar as well as dorsal access is sometimes useful when double plating is necessary (see Fig. 21c, d). If stability cannot be achieved, then an alternative such as spanning plates or external fixation should be used (see Fig. 22c, d).

*Approach* Both dorsal and palmar approaches have to be used, depending on the dislocation and fragments of the fracture.

## Conclusion

A basic understanding of the essential biomechanical characteristics in distal radius fractures seems crucial to achieve sufficient stabilization of the so-called key fragments, thereby avoiding secondary dislocation [51]. The position of the wrist in relation to the radius plays an essential role in distal radius fractures. Furthermore, the palmar and dorsal radio- and ulnocarpal ligaments play an important role in supporting the radius against radial and palmar inclination. Fracture lines are situated between insertion of extrinsic ligaments and form osteoligamentous units which act as key fragments for specific dislocations.

The definition of the so-called key fragments seems to be of particular importance in the restoration of these fractures. If these important parts of the fracture can be identified, fracture-specific stabilization is possible. Due to the ever-increasing number of available implants, fracture-specific restoration and specific plate selection become very relevant. Palmar key fragments should therefore be restored via a palmar access. In case of palmar rim fragments, special hook plates are required to sufficiently capture and stabilize even the smallest pieces. Key fragments on the dorsal side, if large enough, can sometimes be reached indirectly via a palmar access. Dorsal stabilization is indicated for smaller fragments. In case of a central impaction or comminuted fracture of the radius, support of the fracture zone by a grid-like construction of the screws via two distal rows of angle stable screws is essential.

In conclusion, a biomechanical understanding of fracture types leads to a treatment-oriented classification. Recognizing the key fragments leads to a more efficient and specific implant selection. With regard to this finding, the decision making which might be the preferable approach is facilitated.
